# An oncogenic role for sphingosine kinase 2

**DOI:** 10.18632/oncotarget.11714

**Published:** 2016-08-30

**Authors:** Heidi A. Neubauer, Duyen H. Pham, Julia R. Zebol, Paul A.B. Moretti, Amanda L. Peterson, Tamara M. Leclercq, Huasheng Chan, Jason A. Powell, Melissa R. Pitman, Michael S. Samuel, Claudine S. Bonder, Darren J. Creek, Briony L. Gliddon, Stuart M. Pitson

**Affiliations:** ^1^ Centre for Cancer Biology, University of South Australia and SA Pathology, Adelaide, South Australia, Australia; ^2^ School of Biological Sciences, University of Adelaide, Adelaide, South Australia, Australia; ^3^ School of Medicine, University of Adelaide, Adelaide, South Australia, Australia; ^4^ Monash Institute of Pharmaceutical Science, Monash University, Parkville, Victoria, Australia

**Keywords:** sphingosine kinase 2, neoplastic transformation, oncogenesis, proliferation, tumorigenesis

## Abstract

While both human sphingosine kinases (SK1 and SK2) catalyze the generation of the pleiotropic signaling lipid sphingosine 1-phosphate, these enzymes appear to be functionally distinct. SK1 has well described roles in promoting cell survival, proliferation and neoplastic transformation. The roles of SK2, and its contribution to cancer, however, are much less clear. Some studies have suggested an anti-proliferative/pro-apoptotic function for SK2, while others indicate it has a pro-survival role and its inhibition can have anti-cancer effects. Our analysis of gene expression data revealed that SK2 is upregulated in many human cancers, but only to a small extent (up to 2.5-fold over normal tissue). Based on these findings, we examined the effect of different levels of cellular SK2 and showed that high-level overexpression reduced cell proliferation and survival, and increased cellular ceramide levels. In contrast, however, low-level SK2 overexpression promoted cell survival and proliferation, and induced neoplastic transformation *in vivo*. These findings coincided with decreased nuclear localization and increased plasma membrane localization of SK2, as well as increases in extracellular S1P formation. Hence, we have shown for the first time that SK2 can have a direct role in promoting oncogenesis, supporting the use of SK2-specific inhibitors as anti-cancer agents.

## INTRODUCTION

The sphingosine kinases (SKs) catalyze the conversion of sphingosine to sphingosine 1-phosphate (S1P). Given that sphingosine and its precursor, ceramide, are pro-apoptotic molecules, and S1P mediates cell survival and proliferation [1, 2], the SKs are considered critical regulators of the balance between cell death and cell survival, and represent promising targets for anti-cancer therapies [3]. The two mammalian SKs, SK1 and SK2, share high sequence similarity and both possess constitutive catalytic activity, but generally show distinct subcellular localization [4].

The role of SK1 in cancer is well characterized and has been extensively reviewed [1, 2, 5], with high SK1 expression observed in many different cancers and often correlating with poorer patient survival [5]. SK1 overexpression promotes neoplastic transformation and tumorigenesis [6], and notably, targeting SK1 has been shown to attenuate tumor growth in numerous animal models [3]. In contrast, the contribution of SK2 to cancer is unclear. Surprisingly, despite both enzymes catalyzing the same reaction, most studies examining SK2 function have found that it has an opposite role to SK1, and can promote cell cycle arrest and apoptosis [7-11]. Although most of these studies utilized high-level overexpression systems, functional analysis of endogenous SK2 has supported this role in promoting cell death [9, 11, 12]. Most notably, nuclear-localized SK2 has been shown to act as an epigenetic regulator, through S1P-mediated inhibition of histone deacetylase 1/2 (HDAC1/2) activity and increased transcription of p21 and c-fos [13].

Despite this general notion that SK2 is pro-apoptotic, a number of studies have emerged that demonstrate a role for SK2 in promoting cancer. Knockdown of SK2 expression has been shown to enhance apoptosis and chemosensitize many cancer cell types [14-17]. In fact, targeting SK2 in a range of cancer cell lines appears to have more of an anti-cancer effect than targeting SK1 [14, 18]. Strikingly, several *in vivo* studies have reported that targeting SK2 significantly attenuated tumor growth in a range of human xenograft models in mice [19-23]. Increased SK2 expression levels also correlate with disease progression in non-small cell lung cancer (NSCLC) [24] and multiple myeloma [25], and poorer survival in NSCLC patients [24]. Recent work also suggests that SK2 can play a role in increasing telomerase activity [26], promoting the upregulation of c-Myc *via* regulation of HDAC1/2 [20], and facilitating the activation of ezrin-radixin-moesin proteins to promote EGF-induced cancer cell invasion [27], all of which may contribute to cancer development and progression.

Although there is an emerging body of evidence suggesting that SK2 can play a role in cancer development, this is complicated by the known role of SK2 in facilitating cell death, and that, unlike SK1, SK2 overexpression has never been shown to promote neoplastic transformation and tumorigenesis. Here, we demonstrate for the first time that low-level SK2 overexpression, similar to that observed in numerous cancers, can promote cell proliferation, survival and neoplastic transformation, and that these levels of SK2 overexpression alone can drive tumorigenesis *in vivo*.

## RESULTS

### SK2 expression is elevated in a wide range of human cancers

**Figure 1:**
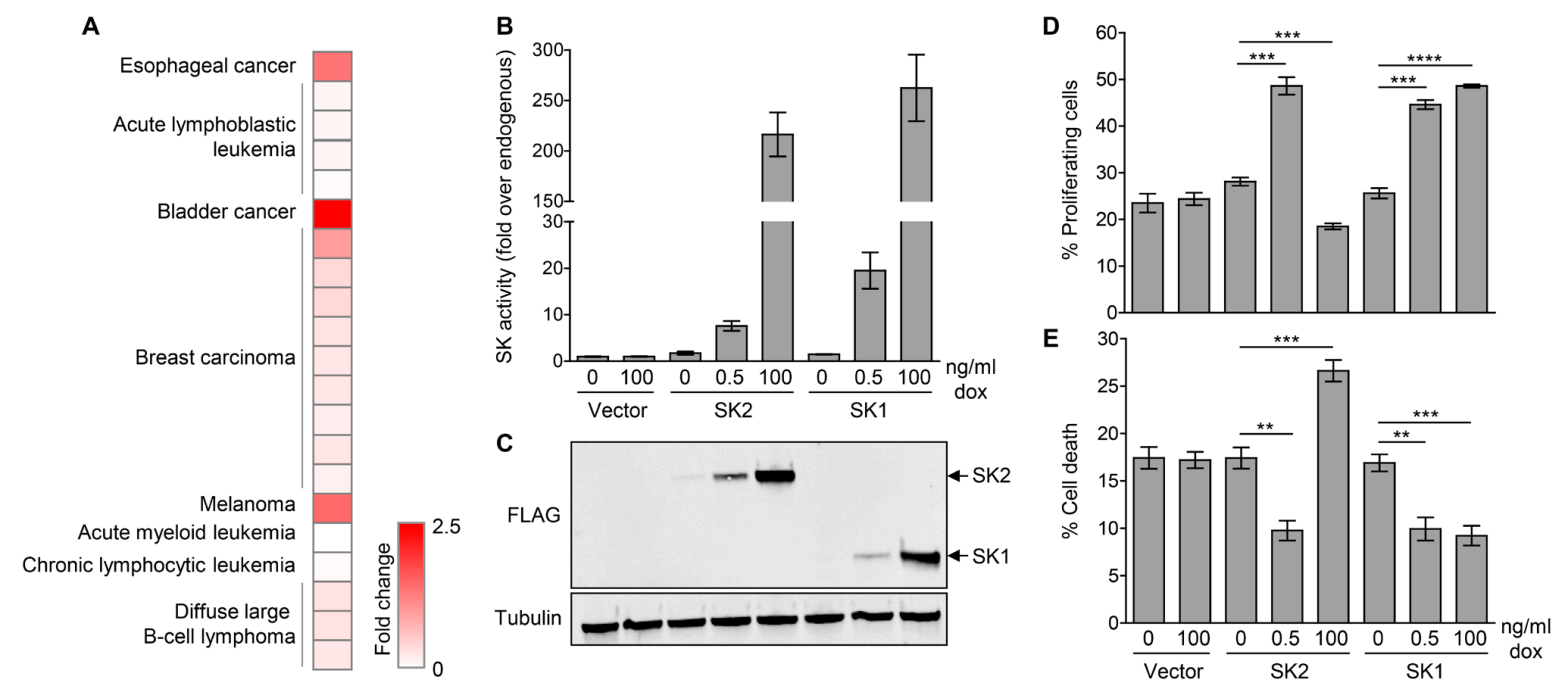
Low-level SK2 overexpression is observed in human cancers, and can promote cell survival and proliferation **A.** Heat map showing human cancers where significant (*p* < 1×10^-4^) upregulation of SK2 mRNA levels have been observed in cancerous tissues compared with corresponding normal tissue. Data was extracted from the Oncomine database [28], where each row represents a cancer subtype from an individual dataset. Further detail is presented in Supplementary Figure S1A. **B.** SK1 and SK2-specific activity upon doxycycline (dox)-induced low- and high-level overexpression in HEK293 Flp-In T-Rex cells. Data shown are mean (± range) of duplicate data points from a representative experiment (of more than three independent experiments). **C.** Lysates from the HEK293 Flp-In T-Rex cells with doxycycline-induced low- and high-level overexpression of FLAG-tagged SK1 or SK2, or empty vector, were subjected to immunoblot analyses with antibodies against FLAG and α-tubulin. Blots shown are representative of at least three independent experiments. **D.** Measurement of cell proliferation in HEK293 Flp-In T-Rex cells with doxycycline (dox)-induced low- and high-level overexpression of SK1 or SK2, or empty vector. Data shown are mean ± SEM, *n* = 3-4. Statistics were performed using an unpaired Student's *t*-test (two-tailed); ***p < 0.001, ****p < 0.0001. **E.** Measurement of cell death in HEK293 Flp-In T-Rex cells with doxycycline (dox)-induced low- and high-level overexpression of SK1 or SK2, or empty vector. Data shown are mean ± SEM, *n* = 4-5. Statistics were performed using an unpaired Student's *t*-test (two-tailed); **p < 0.01, ***p < 0.001.

Despite numerous studies examining the targeting of SK2 in cancer, broad analysis of SK2 expression in cancer has not been previously performed. Thus, we examined SK2 expression in a wide range of human cancers using the public gene expression datasets in the Oncomine database [28]. We found that SK2 is significantly elevated in studies from a broad range of human cancers, including bladder, melanoma, esophageal, breast, lymphoma and leukemia (Figure 1A and Supplementary Figure S1A). Interestingly, however, this cancer-associated elevation in SK2 was only modest, with up to 2.5-fold higher levels of SK2 compared with the corresponding normal tissues. Notably, both SK1 and SK2 were upregulated in three independent datasets for diffuse large B-cell lymphoma (Supplementary Figure S1B), but there was no apparent general correlation between SK2 and SK1 upregulation in the other tumors examined. Indeed, in most other tumor datasets where SK2 was upregulated, SK1 expression was either unaltered or significantly downregulated (Supplementary Figure S1B).

### Low-level SK2 overexpression enhances cell survival and proliferation, whereas high-level overexpression promotes cell death

Many studies have demonstrated that high-level SK2 overexpression can promote cell cycle arrest and cell death [7-10]. However, based on the gene expression analysis showing only low levels of SK2 overexpression in human cancers, we reasoned that more informative functional analysis would be gained by overexpression of SK2 at much lower levels. To investigate this, we utilized human embryonic kidney (HEK) 293 cells engineered to express FLAG-tagged SK2 or SK1 in a doxycycline-inducible, concentration-dependent manner. Using different doxycycline concentrations in the culture media, we could achieve low and high SK2 and SK1 overexpression, as determined by specific-activity (Figure 1B) and protein expression (Figure 1C). Importantly, endogenous SK2 and SK1 protein levels remained unaltered upon induction of SK1 and SK2 overexpression, respectively (Supplementary Figure S2A and S2B). We then used this system to assess the effect of varying levels of SK overexpression on cell proliferation and survival. In agreement with previous studies, overexpression of SK2 at high levels (over 200-fold) in this system resulted in decreased cell proliferation and an increase in cell death (Figure 1D and 1E). Strikingly, however, when SK2 was overexpressed to much lower levels (8-fold over endogenous), more comparable to that seen in the cancer expression analysis, this induced a marked increase in cell proliferation and a decrease in cell death (Figure 1D and 1E). These contrasting findings clearly demonstrate that the cellular levels of SK2 influence its function. Notably, these findings were unique to SK2, with both low- and high-level overexpression of SK1 resulting in a consistent increase in cell survival and proliferation (Figure 1D and 1E).

### SK2 can elicit oncogenic signaling and promote neoplastic transformation *in vitro*

**Figure 2:**
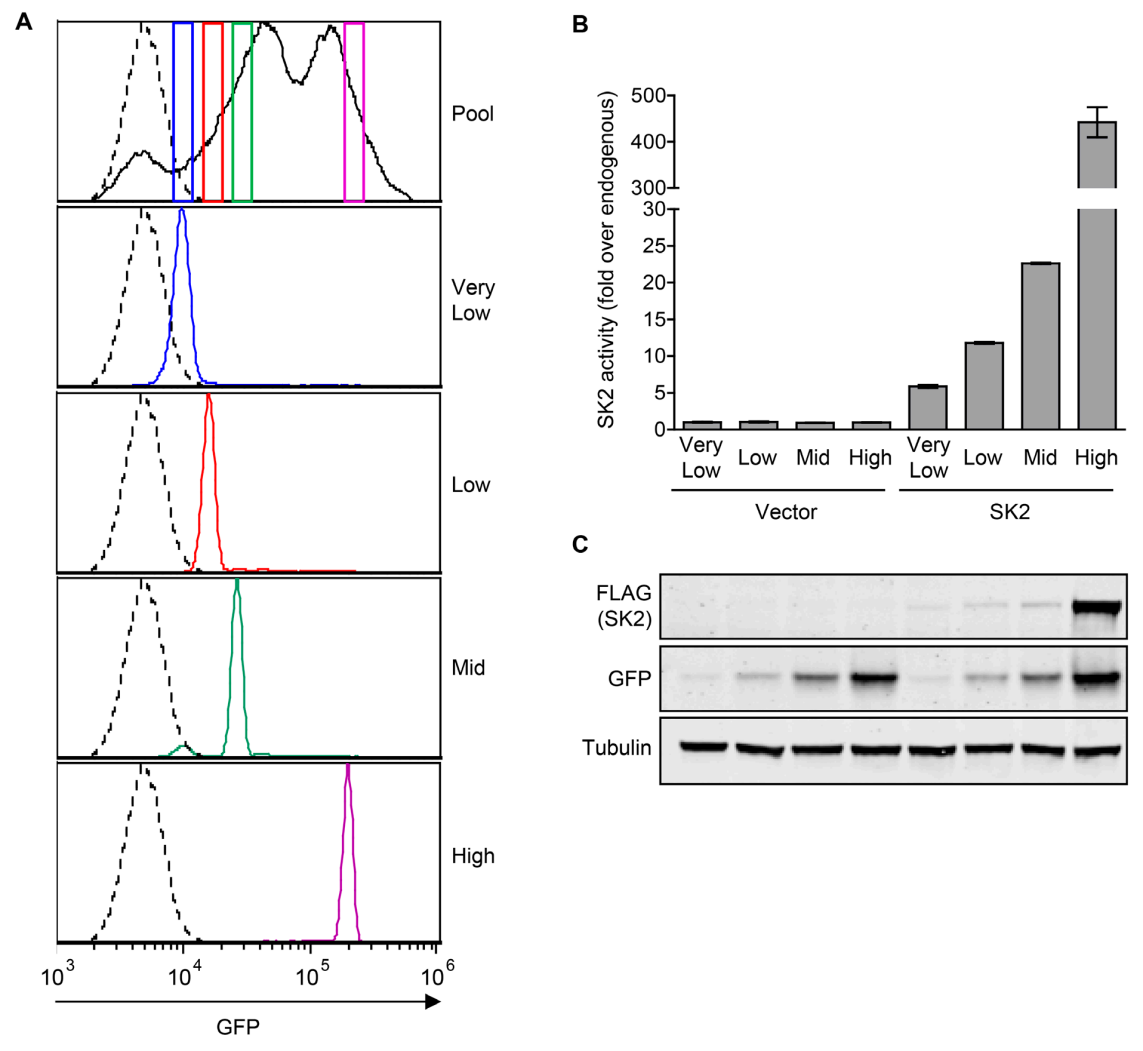
Generation of NIH3T3 stable cell lines with varying levels of constitutive SK2 overexpression **A.** The NIH3T3 pooled stable cell line expressing SK2 and GFP, or GFP alone (empty vector), were sorted on four separate narrow gates of varying GFP intensity (colored boxes in top panel), to produce new stable lines depicted as ‘very low’, ‘low’, ‘mid’ and ‘high’. These new stable lines were then analyzed by flow cytometry to confirm that the desired narrow GFP-expression levels were obtained as expected. GFP-negative control cells are depicted by a dotted line. **B.** SK2-specific activity of NIH3T3 cell lines stably expressing ‘very low’ (5-fold), ‘low’ (10-fold), ‘mid’ (20-fold) or ‘high’ (440-fold) levels of SK2 overexpression (above endogenous levels), or empty vector. Data are shown as mean (± range) of duplicate samples from a representative experiment, of at least three independent experiments. **C.** Lysates from the NIH3T3 vector or SK2 overexpressing cell lines were subjected to immunoblot analyses with antibodies against FLAG, GFP and α-tubulin. Blots shown are representative of at least three independent experiments.

Next, we assessed if low-level overexpression of SK2 could also induce neoplastic transformation, as had been previously observed for SK1 [6]. In contrast to mouse cells, neoplastic transformation of human cells is well known to require multiple oncogenes [29], meaning their use in these type of studies is problematic. Thus, to examine the oncogenic potential of SK2, we transfected NIH3T3 mouse fibroblasts with a vector encoding SK2 as well as green fluorescent protein (GFP) *via* an internal ribosome entry site (IRES) such that GFP and SK2 expression were linked. We then isolated a series of cell lines stably expressing different levels of SK2 through the sorting of cells for differential GFP expression (Figure 2A). The resulting stable cell lines were then validated through the analysis of SK2-specific activity (Figure 2B) and exogenous SK2 protein levels (Figure 2C), which revealed stable overexpression of SK2 at 5-fold, 10-fold, 20-fold and 440-fold over endogenous levels, designated ‘very low’, ‘low’, ‘mid’ and ‘high’ level SK2 overexpression, respectively.

**Figure 3:**
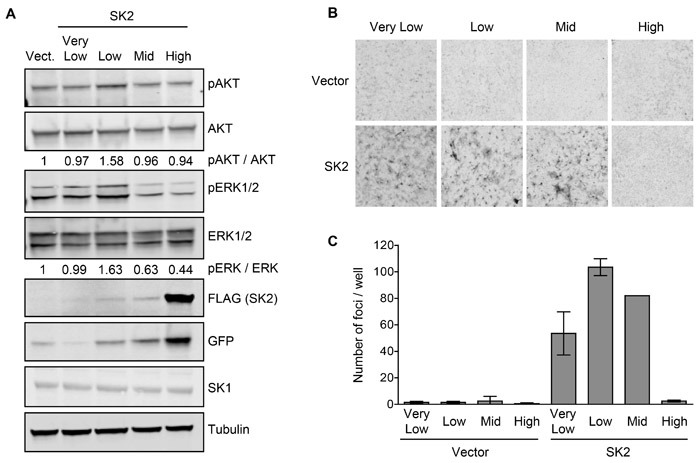
SK2 overexpressed at low levels can elicit oncogenic signaling and drive neoplastic transformation *in vitro* **A.** Lysates from the NIH3T3 vector or SK2-overexpressing cell lines were subjected to immunoblot analyses and probed with antibodies against phospho-AKT, total AKT, phospho-ERK1/2, total ERK1/2, FLAG, GFP, SK1 and α-tubulin. Vect = empty vector with ‘low’ level GFP expression, chosen as a representative control. Densitometry was performed to quantify phospho-AKT and phospho-ERK band intensities, and is presented as a ratio of total AKT and ERK levels, respectively, and is normalized to vector. Blots shown are representative of three independent experiments. **B.** Contact inhibition of the NIH3T3 vector or SK2-overexpressing cell lines was tested using focus formation assays. Images shown are representative of at least three independent experiments, each performed in duplicate, using at least three independently generated sets of stable lines. **C.** Number of foci per well from the experiment shown in Figure 3B were quantified and the mean number of foci for duplicate wells was graphed (± range).

Biochemical analysis of these cell lines revealed that ‘low’ SK2 overexpression resulted in the activation of oncogenic signaling pathways, as demonstrated by an increase in phospho-AKT and phospho-ERK1/2 levels (Figure 3A). Conversely, the ‘mid’ and ‘high’-level SK2 overexpression caused a downregulation of phospho-ERK1/2 signaling (Figure 3A), in agreement with high-level SK2 overexpression attenuating cell proliferation. Also consistent with the HEK293 Flp-In T-Rex overexpression system, endogenous SK1 protein levels were unchanged in the multiple SK2-overexpressing NIH3T3 cell lines, as compared to vector control cells (Figure 3A). These cells were then utilized in *in vitro* assays of neoplastic cell transformation. Notably, in focus formation assays, cell lines with ‘very low’, ‘low’ and ‘mid’-level SK2 overexpression formed foci, whereas cells with ‘high’-level SK2 overexpression did not (Figure 3B and 3C). Similar results were also observed in colony formation assays (Supplementary Figure S3), demonstrating that low-level SK2 overexpression can promote anchorage-independent growth in soft agar. Together, these data demonstrate that low-level SK2 overexpression can elicit oncogenic signaling and induce neoplastic transformation of cells.

### Low-level SK2 overexpression can drive tumor formation *in vivo*

**Figure 4:**
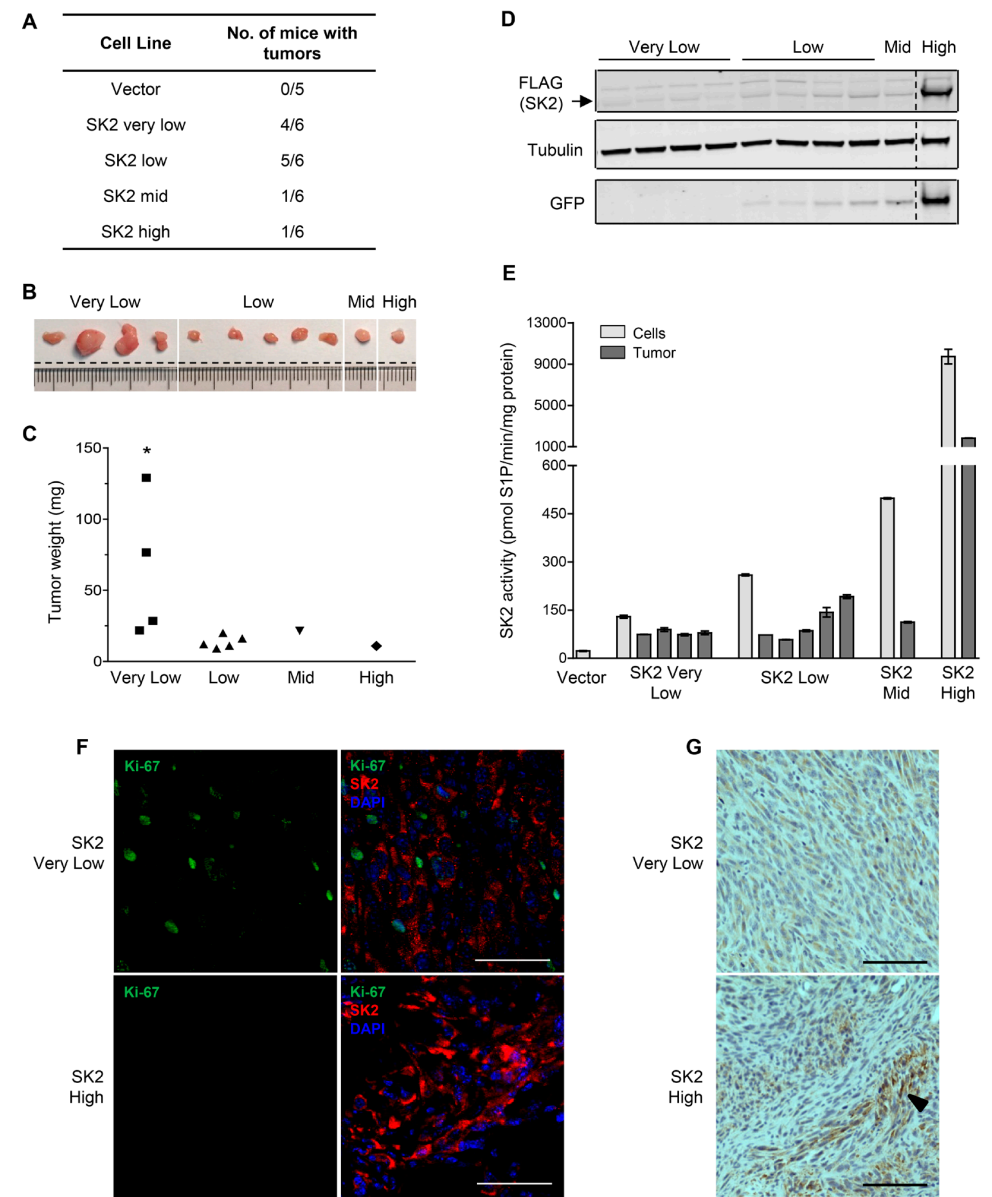
Low-level SK2 overexpression can drive proliferation and tumorigenesis *in vivo* NOD/SCID mice were injected with NIH3T3 cell lines stably overexpressing ‘very low’ (5-fold), ‘low’ (10-fold), ‘mid’ (20-fold) or ‘high’ (440-fold) levels of SK2 (above endogenous levels). Empty vector cells with ‘low’ level GFP expression were chosen as a representative control (Vector). **A.** Table summarizing the number of mice with tumors per cell line, 18 days post-cell injection. **B.** Images of the excised tumors from each group of NIH3T3 stable SK2 cell lines. Dashed lines indicate where the same image has been spliced together to aid interpretation. **C.** Weights of the excised tumors from each group of NIH3T3 stable SK2 cell lines. Statistics denote a significant increase in the weights of SK2 ‘very low’ tumors compared to tumors from the SK2 ‘low’, ‘mid’ and ‘high’ groups (* *p* < 0.05; Student's unpaired two-tailed *t*-test). **D.** Equal amounts of total protein from the tumor tissue lysates were subjected to immunoblot analyses with antibodies against FLAG, α-tubulin and GFP. Each lane represents a different tumor sample. Dashed lines indicate where lanes from the same immunoblots have been spliced together to aid interpretation. **E.** SK2-specific activity from the tumor lysates was measured and graphed as mean (± range) of duplicate samples. These data were plotted alongside data of SK2 activity from the engrafted cell lines, which was transformed from Figure 2B as specific-activity (pmol S1P/min/mg protein) for the purposes of comparison. **F.** Dual immunofluorescence staining of overexpressed FLAG-tagged SK2 (red) and the proliferation marker Ki-67 (green) on the SK2 tumors. Tumor sections were counterstained with DAPI to indicate nuclei (blue). At least five random fields of view were imaged per tumor and representative images are shown. Scale bar = 50 μm. **G.** Levels of SK2 overexpression are heterogeneous in the SK2 ‘high’ tumor. Staining of overexpressed FLAG-tagged SK2 was visualized by immunohistological analyses. Multiple images were taken for each tumor and a representative field of view is shown. Arrow denotes a representative area of heterogeneous, intense FLAG (SK2) staining in the SK2 ‘high’ tumor. Scale bar = 100 μm.

Given that SK2 could promote neoplastic growth *in vitro*, we next examined if this represented full neoplastic transformation through analyzing the ability of these cells to form tumors *in vivo*. Hence, the series of cell lines with differential levels of SK2 overexpression were subcutaneously engrafted into the flanks of NOD/SCID mice, and the development of tumors assessed. Consistent with the *in vitro* data, cells with either ‘very low’ or ‘low’ SK2 overexpression resulted in efficient tumor formation in mice (Figure 4A). In stark contrast, however, cells with either ‘mid’ or ‘high’ SK2 overexpression showed minimal tumor growth (Figure 4A). All tumors were vascularized, as determined by CD31 staining, and showed morphology characteristic of fibrosarcoma (Supplementary Figure S4A and S4B). Notably, the tumors that developed from the ‘very low’ SK2 cells were significantly larger than all other tumors formed (Figure 4B and 4C). Overall, these results demonstrate for the first time that through low-level overexpression, SK2 can drive tumorigenesis *in vivo*.

Although the SK2-expressing cells engrafted into mice were fractionated based on their GFP (and therefore SK2) expression, these cells remained pools of clones. While this obviates potential defects associated with plasmid integration into the genomes of individual clones, it meant that the level of SK2 overexpression observed for each line was an average of all cells within that line. Thus, we examined the expression levels of SK2 within the resulting tumors. Notably, every tumor that developed from the ‘very low’, ‘low’ and ‘mid’ SK2 overexpressing cells all possessed very similar levels of SK2 protein (Figure 4D) and catalytic activity (Figure 4E). Indeed, by comparison to the parental fibroblast cells, it appears that tumor formation resulted preferentially from cells within the pools with less than 5-fold SK2 overexpression (Figure 4E). This finding suggests that this level of SK2 represents the optimal level to promote oncogenic signaling and tumorigenesis, and is consistent with the low level of SK2 upregulation seen in many human cancers (Figure 1A). These results perhaps explain why only one tumor formed from the ‘mid’ SK2-expressing cells, as within this pool of cells there would likely be fewer low SK2-expressing clones compared with the ‘low’ and ‘very low’ groups. Unexpectedly, the ‘high’ SK2-overexpressing cells also resulted in one tumor forming. Further analyses, however, revealed that cells within this tumor were not actively proliferating, whereas cells within the tumors generated from ‘very low’ SK2-expressing cells were highly positive for the proliferation marker Ki-67 (Figure 4F). Furthermore, the level of SK2 overexpression within the ‘high’ SK2 tumor was quite heterogeneous, with only small patches of cells with high levels of SK2 protein (Figure 4G). In fact, the majority of the tumor was comprised of cells with low SK2 overexpression similar in level to that seen in the tumors arising from ‘very low’ SK2-expressing cells (Figure 4G). Therefore, again, it is possible that a small population of cells within the original ‘high’ cell pool drifted to low-level SK2 overexpression and initiated tumor formation, thus supporting the growth of some of the high SK2-expressing cells. Indeed, our data demonstrating that low SK2 overexpression supported enhanced cell proliferation and survival, while high SK2 overexpression had the opposite effect suggests that the high SK2-expressing cells would have been under considerable selective pressure towards low SK2 overexpression.

### Differential levels of SK2 overexpression alter its subcellular localization and sphingolipid metabolism

**Figure 5:**
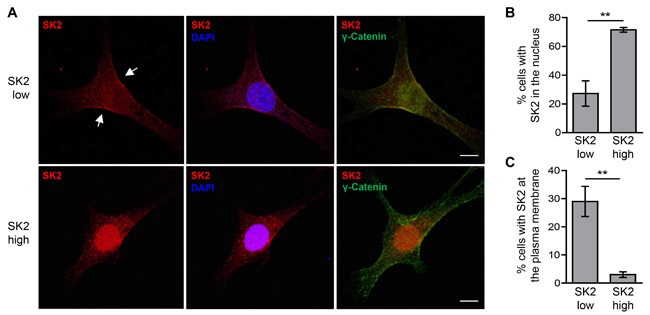
Varying levels of SK2 overexpression affect its subcellular localization **A.** The subcellular localization of FLAG-tagged SK2 (red) in the NIH3T3 stable ‘low’ and ‘high’ SK2-overexpressing cells was examined by immunofluorescence staining, using FLAG antibody. Nuclei were stained with DAPI (blue) and cell membranes were stained with antibodies against γ-catenin (green). Images are representative of cells observed from three independent experiments. Arrows denote representative plasma membrane localization of ‘low’ SK2. Scale bar = 10 μm. **B.**-**C.** Cells from **A.** were visualized by confocal microscopy and scored based on the presence or absence of either **B.** distinct nuclear FLAG-tagged SK2 staining or **C.** plasma membrane-localized FLAG-tagged SK2 staining. A minimum of 200 cells were scored per well, and data were graphed as mean (± SD) of triplicate wells from a single experiment, representative of three independent experiments (** *p* < 0.01; Student's unpaired two-tailed *t*-test).

**Figure 6:**
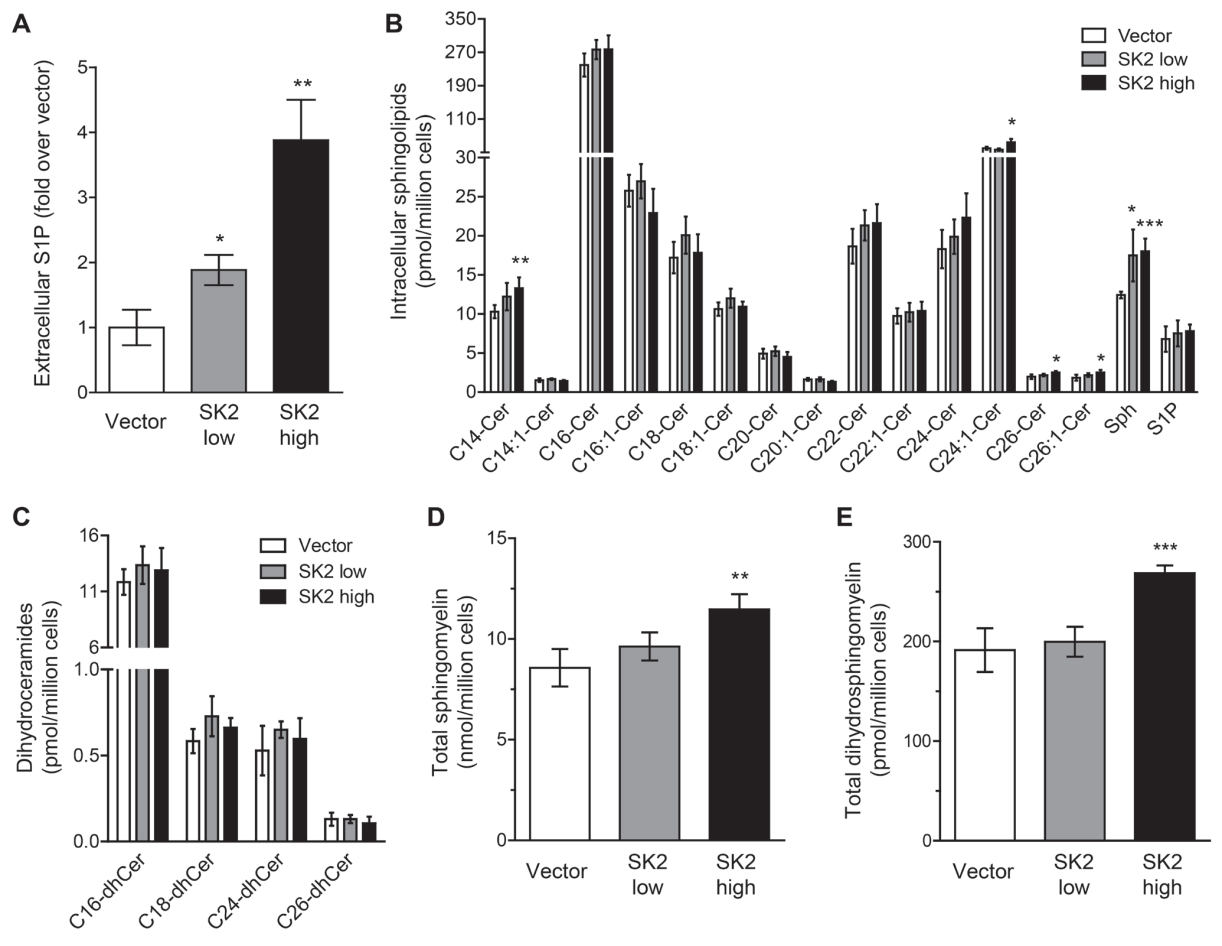
Sphingolipid metabolism is altered when SK2 is overexpressed at varying levels **A.** Rate of extracellular S1P formation was determined from intact vector control ‘low’, SK2 ‘low’ and SK2 ‘high’ NIH3T3 stable cell lines. Analyses were performed in triplicate and data are graphed as mean (± SD). Statistics denote significant increases in extracellular S1P compared to vector control cells (* *p* < 0.05, ** *p* < 0.01; Student's unpaired two-tailed *t*-test). **B.**-**E.** Intracellular sphingolipid species were analyzed by LC-MS using NIH3T3 vector control ‘low’, SK2 ‘low’ and SK2 ‘high’ stable cell lines. Data are graphed as mean (± SD) of quadruplicate samples for **B.** individual ceramide species, sphingosine (Sph) and sphingosine 1-phosphate (S1P), **C.** individual dihydroceramide species, **D.** total sphingomyelin levels, and **E.** total dihydrosphingomyelin levels. Statistics denote significant increases in lipids compared to vector control cells (* *p* < 0.05, ** *p* < 0.01, *** *p* < 0.001; Student's unpaired two-tailed *t*-test).

It is well established that the subcellular localization of the SKs, and hence the compartmentalization of S1P within the cell, plays an important role in the function of these enzymes [30]. The oncogenic role of SK1 requires its translocation to the plasma membrane, a location that results in increased extracellular S1P production [31]. Furthermore, the localization of SK2 to the nucleus, endoplasmic reticulum (ER) or mitochondria appears to promote its anti-proliferative, pro-apoptotic functions [7, 8, 11, 13]. Thus, we examined the localization of SK2 when overexpressed at low and high levels. In the ‘high’ SK2-expressing cells, SK2 was strongly nuclear-localized (Figure 5A and 5B), in agreement with previous reports for this cell type [7]. In the ‘low’ SK2-expressing cells, however, SK2 was mostly cytoplasmic, and showed a significant increase in its plasma membrane localization (Figure 5A and 5C). Furthermore, the formation of extracellular S1P was significantly higher from cells with ‘low’-level SK2 overexpression compared to vector control cells (Figure 6A), consistent with our observations of increased SK2 at the plasma membrane in these cells. Interestingly, ‘high’-level SK2 overexpression resulted in a further increase in extracellular S1P formation (Figure 6A), but this was only a modest doubling compared with the ‘low’ SK2 cells, despite these cells having greater than 400-fold more SK2 activity.

Sphingolipid analysis revealed, somewhat surprisingly, that ‘low’-level SK2 overexpression had very little effect on the intracellular levels of S1P, ceramides, dihydroceramides, sphingomyelins or dihydrosphingomyelins (Figure 6B-6E), with the only change noted being a small increase in sphingosine. In contrast, ‘high’-level SK2 overexpression resulted in a significant increase in a range of ceramide species, sphingomyelins and dihydrosphingomyelins, as well as sphingosine (Figure 6B, 6D and 6E), in line with a previous report demonstrating that overexpressed SK2 partially localized to the ER, and S1P produced here could feed into an ER/golgi-associated ‘salvage pathway’ to generate pro-apoptotic sphingosine and ceramide, as well as sphingomyelin [8]. The increase in ceramides and sphingosine are likely to contribute, at least in part, to the anti-proliferative and pro-cell death role of SK2 in these ‘high’ overexpression cells, with the increase in extracellular S1P formation that was observed in these cells (Figure 6A) possibly insufficient to override these effects.

## DISCUSSION

Despite the conflicting data in the literature, our findings demonstrate that, like SK1, SK2 can have a physiological role in promoting cell survival and proliferation, potentially through plasma membrane localization. This is perhaps not surprising, as the individual genetic deletion of either SK1 or SK2 in mice does not result in any gross phenotypic abnormalities [32, 33], whereas the double knock-out mice die *in utero* [34], suggestive of at least some functional redundancy between the two proteins. Like SK1, there are no mutations in SK2 linked to cancer, however it has been suggested that cancer cells can display a ‘non-oncogene addiction’ for SK1 [2, 35]. Given that we have now shown that SK2 can promote neoplastic transformation and tumorigenesis, and is upregulated in many human cancers, coupled with the anti-cancer efficacy of SK2-selective inhibitors leads us to postulate that a non-oncogene addiction may apply for both SKs in cancer. Indeed, targeting both SK isoforms may be the best strategy to overcome tissue and cell type-specific differences in the roles of the SKs in different cancers, and in agreement, dual SK1/SK2 inhibitors show significant decreases in tumor burden *in vivo* [36, 37].

One of the most intriguing findings of our study is the observation that SK2 function can dramatically switch, depending on its expression level, from being pro-survival and pro-proliferative to pro-cell death and anti-proliferative. While it could be argued that high-level overexpression is non-physiological, and may generate artefacts, it is notable that previous studies have shown that SK2 can have physiological roles in promoting cell cycle arrest and apoptosis when localized to organelles such as the nucleus and mitochondria [11, 13]. Clearly, under normal conditions these pro-death roles are likely kept under tight regulation, so it remains possible that high-level overexpression circumvents these regulatory mechanisms. This high-level overexpression of SK2, for example, may lead to altered protein-protein interactions or post-translational modifications, altering the subcellular localization of SK2, from the plasma membrane to cellular organelles such as the nucleus, in favor of promoting cell death. Indeed, it has been previously reported that when transiently overexpressed, SK2 could interact with and sequester the pro-survival Bcl-x_L_ protein [10], suggesting a possible mechanism for the pro-apoptotic phenotype we observed with high-level SK2 overexpression. However, despite multiple attempts, we were unable to detect an interaction between Bcl-x_L_ and SK2 in our system, suggesting that this proposed interaction was unlikely to mediate the observed phenotype. Intriguingly, there appears to be a window whereby low-level SK2 upregulation confers a survival and proliferative advantage to the cell without inducing these pro-apoptotic functions. Whether a different subset of post-translational modifications and/or protein-protein interactions drive the differences in subcellular localization and function observed with low *versus* high SK2 overexpression will require further interrogation.

To add further complexity, other studies have demonstrated that nuclear SK2 can contribute to cancer progression through the stabilization of telomerase and promotion of c-Myc expression [20, 26]. In the present study, SK2 was observed in the nucleus when overexpressed at high levels, and yet here it had an opposite, anti-proliferative role, which is also well documented [7, 13]. It is unclear how SK2 can have such vastly different functions within the same organelle, but it suggests that there must be additional factors regulating these processes. Indeed, the regulatory mechanisms controlling this enzyme remain an important, but currently largely unanswered question [4]. Notably, SK1 remains pro-survival and pro-proliferative even at high-level overexpression, likely due to its different subcellular localization to SK2 and consequent contribution to different sphingolipid pools within the cell. In line with this, previous studies have shown that artificially targeting SK1 to the ER can render it pro-apoptotic, like SK2 [8].

Interestingly, while cells with 20-fold (‘mid’) SK2 overexpression were able to form foci *in vitro*, they had decreased levels of phospho-ERK1/2 and did not efficiently form tumors *in vivo*. This may indicate that this level of SK2 overexpression is at the upper limit of the ‘window’ whereby SK2 switches from being tumourigenic to having predominantly anti-proliferative functions. Conversely, 5-fold (‘very low’) SK2 overexpression resulted in no appreciable changes in phospho-ERK1/2 or phospho-AKT levels, and yet these cells developed the largest tumors *in vivo*. It is therefore possible that S1P-mediated angiogenesis and tumor vascularization played more of an important role in the development of these tumors, given that low-level overexpression of SK2 resulted in plasma membrane localization and increased formation of extracellular S1P, which is a key regulator of angiogenesis [38]. These observed differences also highlight the importance of employing *in vivo* models for assessment of full neoplastic transformation. Furthermore, it was surprising that ‘high’-level SK2 overexpression resulted in a doubling in extracellular S1P production as compared to the ‘low’-SK2 overexpressing cells, despite ‘high’ SK2 overexpression resulting in decreased survival and proliferative signaling, and increases in pro-apoptotic sphingolipid species. Notably, Weigert *et al*. previously reported that transient overexpression of SK2 resulted in a substantial increase in S1P released from apoptotic cells as a result of SK2 being cleaved by caspase-1 and secreted from the cell [39]. This may, in part, explain our observed increase in extracellular S1P in the SK2 ‘high’ cells, which was clearly not able to facilitate any overall pro-survival or proliferative stimulus in these cells. It should be noted that Liang *et al*. found a significant increase in colitis-associated tumor development in SK2 knockout mice, when compared to wildtype mice [40], suggesting that SK2 may function as a tumor suppressor in this model. However, these findings are likely to be an indirect effect as it was also shown in the study that the global genetic loss of SK2 caused an upregulation of both S1P receptor 1 and SK1 levels in the colon, with a concomitant increase in circulating and colonic S1P [40]. SK1 has been previously shown to contribute to colon carcinogenesis [41] and indeed, the increase in severity of colitis in the SK2 knockout mice was ablated by the SK1-specific inhibitor SK1-I [40]. Furthermore, the proposed tumor-suppressive role of SK2 in negatively regulating pro-tumorigenic SK1 levels is not recapitulated with SK2-selective inhibitors, which show efficacy in decreasing tumor burden in murine xenograft models [20-23].

Evidently the true functions of SK2 are complex and are also likely to be tissue- and cell type-specific. However, from our findings it is clear that SK2 represents an important target in cancer and future work to better understand how SK2 is regulated will be important for the generation of more efficacious SK2-targeting anti-cancer drugs.

## MATERIALS AND METHODS

### Antibodies

The following primary antibodies were utilized: mouse monoclonal anti-FLAG (Clone M2 #F3165, Sigma-Aldrich), rabbit polyclonal anti-FLAG (#2368, Cell Signaling Technology), rabbit monoclonal anti-FLAG (#14793, Cell Signaling Technology), mouse anti-α-tubulin (#ab7291, Abcam, Cambridge, MA, USA), goat anti-GFP (#600-101-215, Rockland Immunochemicals, Limerick, PA, USA), rabbit anti-SK1 (#SP1621, ECM Biosciences, Versailles, KY, USA), rabbit anti-SK2 (#17096-1-AP, Proteintech, Rosemont, IL, USA), rabbit anti-phospho-p44/42 MAPK (ERK1/2) Thr202/Tyr204 (#9101, Cell Signaling Technology), rabbit anti-p44/42 MAPK (ERK1/2) (#9102, Cell Signaling Technology), rabbit anti-phospho-AKT Ser473 (#9271, Cell Signaling Technology), rabbit anti-AKT (#9272, Cell Signaling Technology), mouse anti-Ki-67 (#VP-K452, Vector Labs, Burlingame, CA, USA), mouse anti-γ-Catenin (#610253, BD Biosciences) and goat anti-PECAM-1 (CD31; #SC-1506, Santa Cruz Biotechnology, Dallas, TX, USA).

### Generation of cell lines

HEK293 Flp-In T-Rex cells (Invitrogen, Life Technologies) with doxycycline-inducible FLAG-tagged SK1 or SK2 expression, or empty vector, were generated as previously described [42].

To generate NIH3T3 cells with varying levels of constitutive SK2 overexpression, we obtained a pCX-EGFP construct [43] that we initially modified by replacing the EGFP with a polylinker following digestion with EcoRI and ligation of annealed kinased oligonucleotides AATTCGG TACCGAGCTCGCTAGCGCGGCCGCCTCGAGC-3′ and 5′-AATTGC TCGAGGCGGCCGCGCTAGCGAGCTCGGTACCG-3′ to produce pCX4. pCX4-IRES EGFP was then generated by subcloning the IRES EGFP cassette from pcDNA3-IRES EGFP [44] with EcoRI and NotI. pCX4-SK2(FLAG) IRES EGFP was then made by cloning in FLAG-tagged human SK2a [45] following digestion with EcoRI. NIH3T3 mouse fibroblasts (ATCC CRL-1658) were transfected with pCX4-SK2(FLAG) IRES EGFP, or empty vector, using Lipofectamine™ 2000 (Invitrogen) as per the manufacturer's protocol. 48 h after transfection, the cells were sorted for GFP-positive cells using a MoFlo Astrios cell sorter (Beckman Coulter). A stable GFP-positive cell population was obtained by sorting for GFP another two times. The stable GFP-positive pooled line was then sorted on four separate narrow gates of varying GFP intensity, to produce new stable lines depicted as ‘very low’, ‘low’, ‘mid’ and ‘high’. These new stable lines were then analyzed by flow cytometry to confirm that the desired narrow GFP-expression levels were obtained as expected.

### Cell culture

HEK293 Flp-In T-Rex cells were cultured in Dulbecco's modified Eagle's medium (Gibco, Invitrogen), containing 10% fetal bovine serum (Bovagen), 1 mM HEPES, penicillin (1.2 mg/ml) and streptomycin (1.6 mg/ml). NIH3T3 mouse fibroblasts were cultured in Dulbecco's modified Eagle's medium (Gibco, Invitrogen), containing 10% donor bovine serum with iron (DBS; Gibco, Invitrogen), 1 mM HEPES, penicillin (1.2 mg/ml) and streptomycin (1.6 mg/ml). All cells were grown at 37°C, 5% CO_2_ in a humidified incubator.

For doxycycline-induced low- and high-level overexpression of FLAG-tagged SK1 and SK2, HEK293 Flp-In T-Rex cells were seeded, and 24 h later media was removed and replaced with serum-free media containing 0.1% bovine serum albumin (BSA; Sigma Aldrich) with either vehicle (methanol, 0.001% v/v final), 0.5 ng/ml or 100 ng/ml doxycycline (Sigma Aldrich). After 24 h induction, cells were harvested and lysates prepared. Cell proliferation and cell death were determined as previously described [36].

### Immunoblot analysis

Cells were harvested by scraping into cold phosphate buffered saline (PBS), and cell pellets were resuspended in extraction buffer (50 mM Tris-HCl buffer (pH 7.4) containing 150 mM NaCl, 10% glycerol, 1 mM EDTA, 0.05% Triton X-100, 2 mM Na_3_VO_4_, 10 mM NaF, 10 mM β-glycerophosphate, 1 mM dithiothreitol and protease inhibitor cocktail (Roche)). Cells were lysed by sonication, and total protein concentration was determined by a Bradford protein assay (Bio-Rad Laboratories, Hercules, CA, USA). Lysates of equal protein were separated by SDS-PAGE on a Criterion™ XT Bis-Tris 4-12% gradient gel (Bio-Rad Laboratories) under reducing conditions. Proteins were transferred to nitrocellulose membrane (Pall Life Sciences, Pensacola, FL, USA). Membranes were blocked using Odyssey® Blocking Buffer (LI-COR, Lincoln, NE, USA), and subjected to immunoblotting with various primary antibodies. Proteins were visualized using IRDye® secondary antibodies and the Odyssey® CLx infrared imaging system (LI-COR). Densitometry was performed using ImageQuant 5.2 analysis software (Molecular Dynamics).

### Focus formation assays

Focus formation assays were performed as described previously [6]. Briefly, cells were cultured to form monolayers in 6-well plates in DMEM with 1% DBS (Gibco, Life Technologies), and media was replenished every 2-3 days for a total of 3 weeks. Cells were then fixed in methanol, foci were stained with bromophenol blue, and images were taken using the Odyssey® CLx infrared imaging system (LI-COR).

### Colony formation in soft agar

Colony formation assays in soft agar were performed as previously described [36]. After 14-21 days, colonies were quantified visually using light microscopy. Colonies were imaged using an Olympus MVX10 microscope.

### In vivo tumor model

Experiments involving mice were conducted according to the guidelines from the Australian code of practice for the care and use of animals for scientific purposes 7th Edition, 2004, and with approval from the SA Pathology/CALHN Animal Ethics Committee and the University of Adelaide Animal Ethics Committee.

The NIH3T3 cell lines expressing varying levels of SK2 described above were trypsinized and washed in PBS, and 1×10^6^ cells were injected in 200 μl of PBS subcutaneously into the flank of 8 week old female NOD/SCID mice. The empty vector cells with ‘low’ GFP expression were selected as a representative control group. Mice were examined daily to monitor tumor formation. On day 19, all mice were humanely killed and tumors were excised. Half of each tumor was fixed in 10% formalin, paraffin embedded and sectioned. The remaining half was homogenized using a pestle (Axygen) in extraction buffer, subjected to freeze/thawing in liquid nitrogen and sonication, and lysates were clarified by centrifugation at 17,000 × g for 15 min at 4°C.

### Sphingosine kinase activity assays

SK1 and SK2 activity was determined as previously described, using isoform-selective assay conditions [46].

### Immunohistological analyses

Immunohistological staining was performed as previously described [36], with the following modifications. For PECAM-1 (CD31) expression, sections were blocked with rabbit serum, incubated with goat polyclonal antibody to PECAM-1 (Santa Cruz Biotechnology) at 133 ng/ml, and biotinylated rabbit anti-goat secondary antibody (1:500; Abcam). For FLAG-tagged SK2 expression, sections were blocked with goat serum, incubated with rabbit polyclonal FLAG antibody (Cell Signaling Technology) at 150 ng/ml, and biotinylated goat anti-rabbit secondary antibody (1:500; Vector Labs). Sections were visualized on an EVOS XL light microscope (Life Technologies) at 20x magnification.

### Immunofluorescence analyses

For dual immunofluorescence staining of overexpressed FLAG-tagged SK2 and Ki-67 on the formalin-fixed paraffin-embedded tumor tissue samples, sections were de-waxed, rehydrated, and antigen retrieval was performed by boiling in citrate buffer for 30 min. Sections were blocked in 10% goat serum diluted in CAS-Block (Thermo Fisher Scientific) for 30 min. Following blocking, sections were incubated overnight at 4°C with mouse monoclonal anti-Ki-67 antibody (1:20; Vector Labs) and rabbit polyclonal anti-FLAG antibody (1:100; Cell Signaling Technology) diluted together in 10% goat serum/CAS-Block. Sections were then incubated for 1 h at room temperature with goat anti-mouse AlexaFluor 488 (1:400) and goat anti-rabbit AlexaFluor 594 (1:400) secondary antibodies (Thermo Fisher Scientific) diluted together in 10% goat serum/CAS-Block. Labelled sections were then mounted in Vectashield mounting medium containing DAPI (Vector Labs) and were imaged using a Zeiss LSM 700 confocal microscope (Jena, Germany).

To examine the subcellular localization of overexpressed FLAG-tagged SK2, NIH3T3 SK2 ‘low’ and ‘high’ stable cell lines were seeded onto 8-well glass chamber slides (Nalge Nunc International) coated with poly-L-lysine (Sigma-Aldrich) at 4×10^4^ cells/well, and grown overnight in DMEM with 10% DBS. Media was then removed and replaced with DMEM containing 0.5% DBS, and cells were cultured for a further 16 h. Cells were then fixed in 4% paraformaldehyde for 10 min, permeabilized for 10 min in PBS with 0.1% Triton X-100, and blocked in 5% goat serum in PBS with 0.1% Triton X-100 for 60 min. Rabbit monoclonal anti-FLAG antibody (1:200; Cell Signaling Technology) and mouse anti-γ-catenin antibody (1:500; BD Biosciences) were incubated for 1.5 h at room temperature, followed by goat anti-rabbit AlexaFluor 594 and goat anti-mouse AlexaFluor 488 secondary antibodies (1:500; Thermo Fisher Scientific) for 1 h. Cells were counterstained with DAPI to identify nuclei (blue). Fluorescence microscopy and imaging were performed using a Zeiss LSM 700 confocal microscope.

### Measuring rate of extracellular S1P production

Rate of extracellular S1P formation from intact vector control ‘low’, SK2 ‘low’ and SK2 ‘high’ NIH3T3 stable cell lines was determined essentially as previously described [47]. Briefly, cells were seeded in equal numbers at 80% confluence (in 20 cm^2^ dishes), and media was replaced with DMEM containing 0.5% DBS and incubated for a further 16 h. Cells were then labelled with 0.5 μCi of [^3^H]-sphingosine (Perkin-Elmer, Rowville, VIC, Australia) for 30 min, after which the conditioned media was collected. Extracellular [^3^H]-S1P generated in the conditioned medium was extracted and analyzed by scintillation counting.

### Intracellular sphingolipid analyses

Vector control ‘low’, SK2 ‘low’ and SK2 ‘high’ NIH3T3 stable cell lines were grown in DMEM with 10% DBS to 80% confluence, media was replaced with DMEM containing 0.5% DBS and cells were cultured for 16 h. Cells were trypsinized, quenched and washed in PBS. Cells were pelleted in quadruplicate with 8.8×106 cells per sample, and intracellular sphingolipid species were analyzed by LC-MS, as previously described [48] with the following minor modifications. Prepared samples were injected onto an Ascentis Express C18 column (Supelco Analytical, Bellefonte, PA, USA), and non-natural sphingolipid internal standards were added to each sample to allow relative quantification. Data analysis was performed using Tracefinder (Thermo Fisher Scientific).

## SUPPLEMENTARY MATERIAL FIGURES


