# Supplementary material for: An oncogenic role for sphingosine kinase 2
Source: Oncotarget. 2016 Aug 30;7(40):64886–99. doi: 10.18632/oncotarget.11714 (PMC5323123; doi:10.18632/oncotarget.11714)
Supplement: Supplementary file 1 [file oncotarget-07-64886-s001.pdf]

An oncogenic role for sphingosine kinase 2

Supplementary Material

A

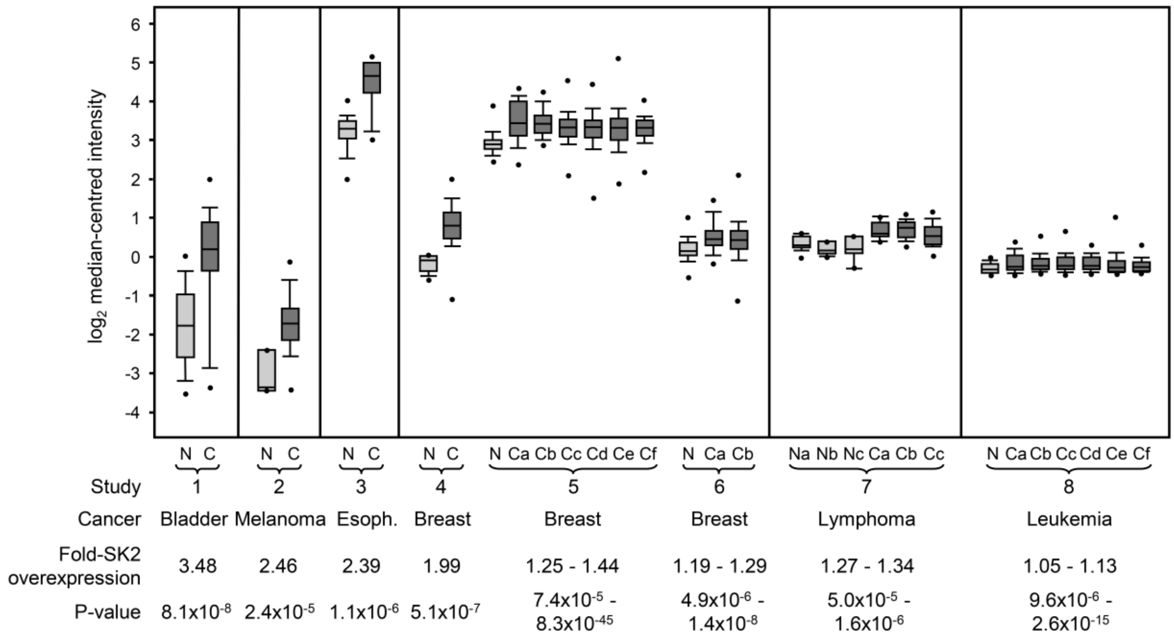

B

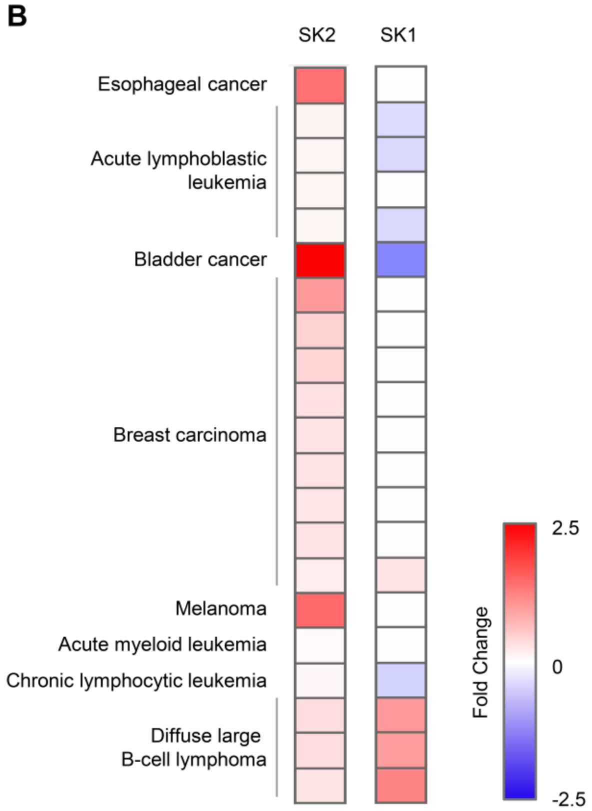

Supplementary Figure S1: SK2 is upregulated in human cancers. (A) Box plots showing human cancers with significant upregulation of SK2 mRNA levels. N = normal tissue; C = cancerous tissue. Data were extracted from the Oncomine database, from the following studies: study 1, Sanchez-Carbayo Bladder 2; study 2, Talantov Melanoma; study 3, Kim Esophagus; study 4, Finak Breast; study 5, Curtis Breast; study 6, TCGA Breast; study 7, Compagno Lymphoma; study 8, Haferlach Leukemia. **(B)** Heat map showing significant (p<1x10^{-4}) changes in SK1 mRNA levels in the human cancer datasets described above, as compared with corresponding normal tissues.

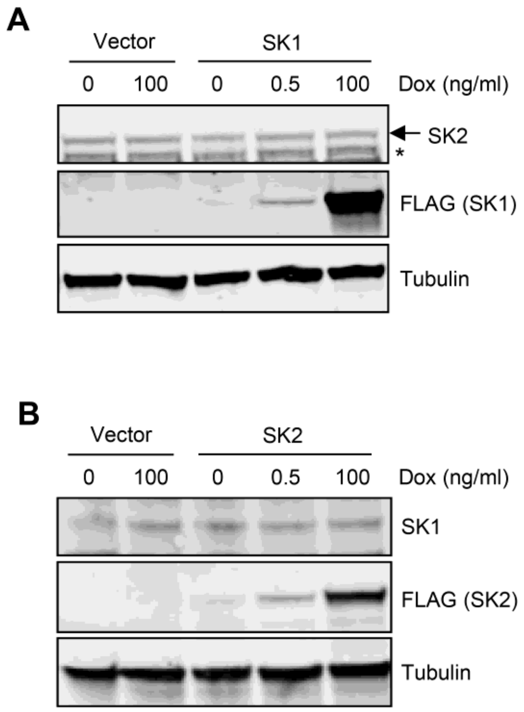

Supplementary Figure S2: Endogenous SK protein levels remain unchanged in the HEK293 Flp-In T-Rex overexpression systems. (A) Western blot analysis of endogenous SK2 protein levels in lysates from HEK293 Flp-In T-Rex cells with doxycycline (dox)-induced low- and high-level FLAG-tagged SK1 overexpression, or vector control. Asterisk (*) denotes a non-specific band. Blots shown are representative of three independent experiments. **(B)** Western blot analysis of endogenous SK1 protein levels in lysates from HEK293 Flp-In T-Rex cells with doxycycline (dox)-induced low- and high-level FLAG-tagged SK2 overexpression, or vector control. Blots shown are representative of three independent experiments.

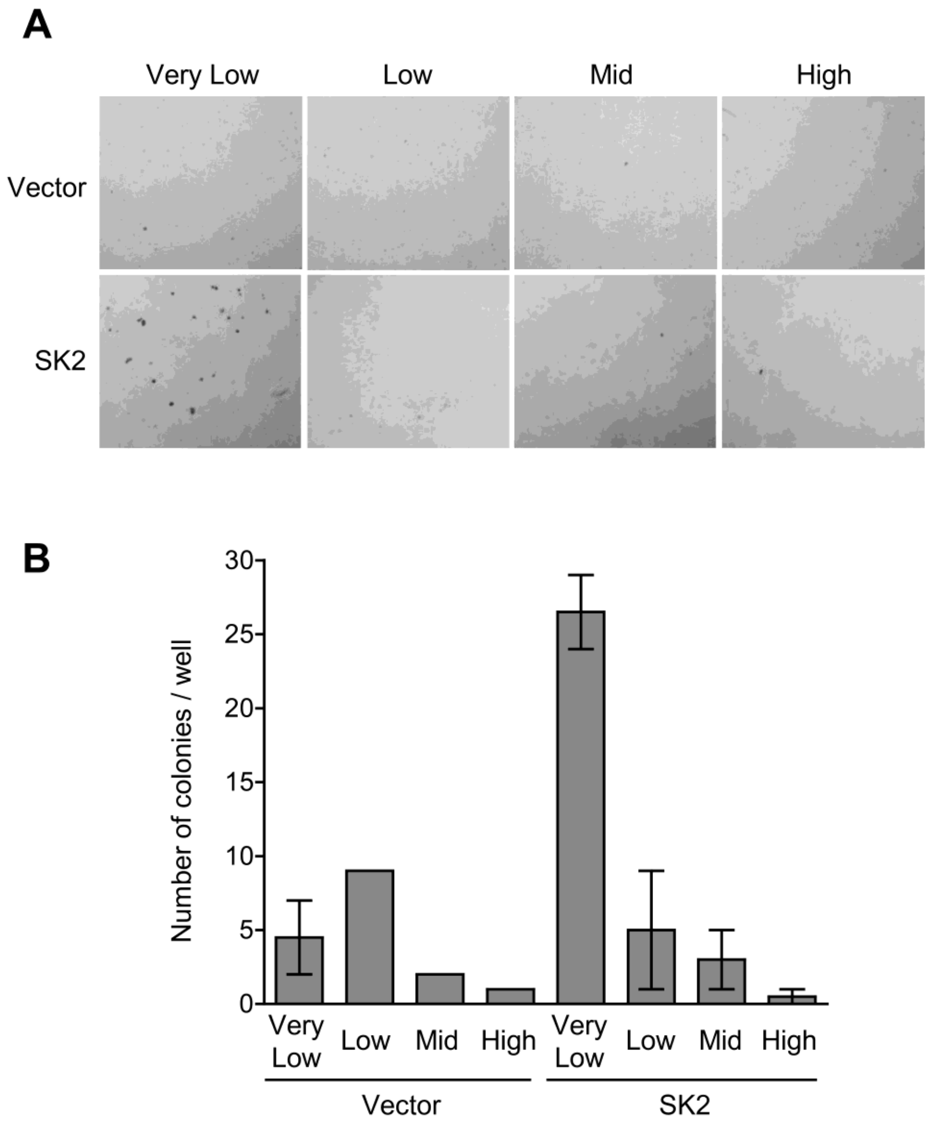

Supplementary Figure S3: Low-level SK2 overexpression can promote anchorage-independent growth of cells. Anchorage-independent growth of the NIH3T3 empty vector or SK2-overexpressing cell lines was tested using colony formation in soft agar. Representative images of colonies formed are shown **(A)** and quantified (mean ± range) **(B)**.

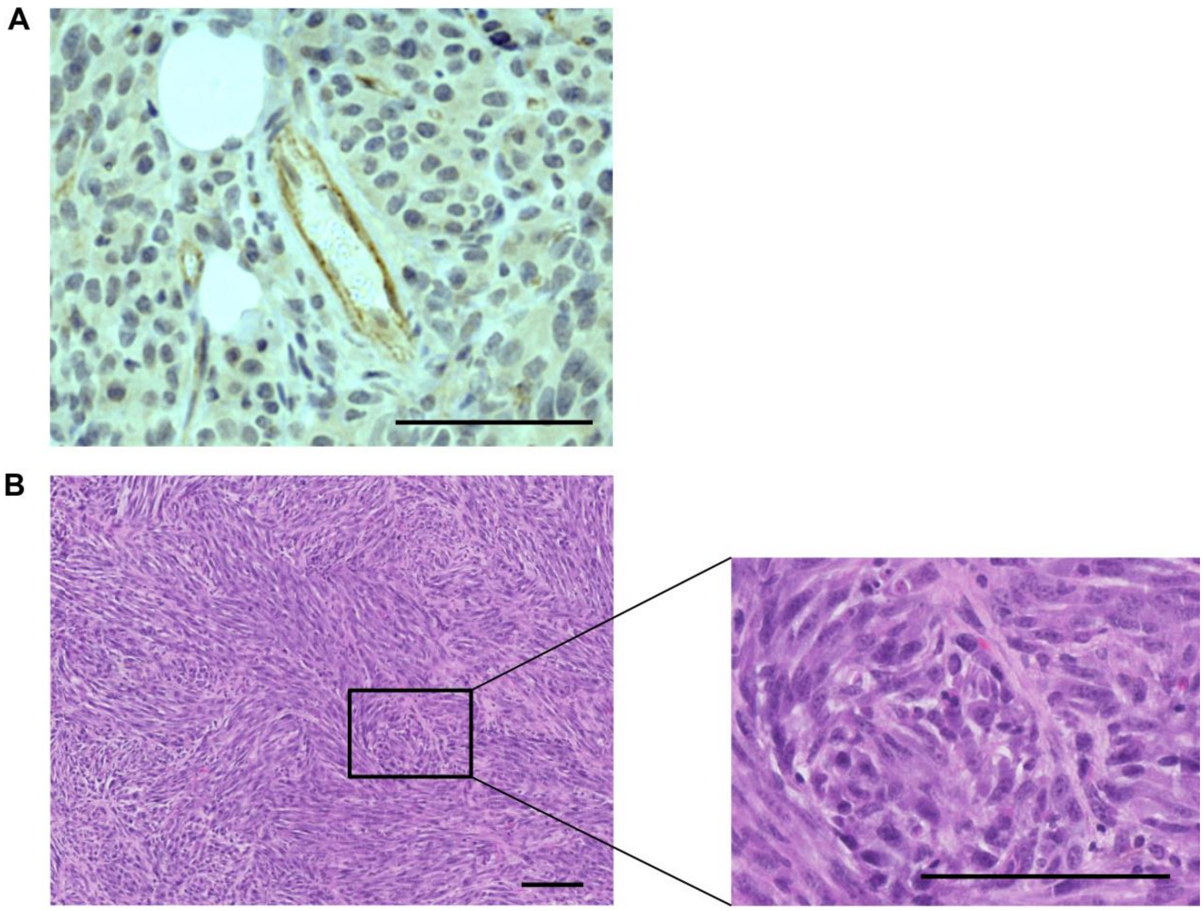

Supplementary Figure S4: Tumors formed from the transformation of NIH3T3 mouse fibroblasts overexpressing SK2 are vascularized and characteristic of fibrosarcoma. (A) The presence of vasculature within the tumor tissue sections was examined by PECAM-1 (CD31) staining. A representative image is shown at 20x magnification. Scale bar = 100 µm. **(B)** Morphology of the tumor tissue sections is shown by hematoxylin and eosin staining. The image on the right is a 20x magnification of the area framed in the left (5x magnification) image. Scale bar = 100 µm.
